# Making sense of it all: Ethical reflections on the conditions surrounding the first genome-edited babies

**DOI:** 10.12688/wellcomeopenres.16295.1

**Published:** 2020-09-16

**Authors:** Qi Chen, Yonghui Ma, G Owen Schaefer, Vicki Xafis, Markus Labude, Peter Mills

**Affiliations:** 1 Centre for Bioethics, Medical School, Xiamen University, Xiamen, 361102, China; 2 Centre for Biomedical Ethics, National University of Singapore, Singapore, 117597, Singapore; 3 Nuffield Council on Bioethics, London, WC1B 3JS, UK

**Keywords:** He Jiankui, Human germline genome editing, CRISPR-Cas9, HIV, Gene editing, Twins/genetics, Research ethics, Research governance

## Abstract

In November 2018 the birth of the first genome-edited human beings was announced. The ensuing ethical controversy, institutional investigations and legal proceedings led to the revision of standards, rules and procedures at many levels. Arguably, however, these developments have not fundamentally changed the conditions or the culture that nourished He Jiankui’s vaulting ambition in the first place and enabled it to find expression. In this paper we explore the clinical, regulatory and societal circumstances of the ‘gene-edited baby’ case, the political, cultural and economic conditions that created a radical and dangerous climate for biotechnology innovation, and the responsibilities of the international research community, many of whose members were apprised of Dr He’s intentions. The aim is not to heap anathemas on the heads of implicated individuals but to draw attention to the need for different communities (researchers, authorities and domestic publics) actively to play a part in the governance of biomedical innovation and for research to be bridled by human values.

## Disclaimer

The views expressed in this article are those of the author(s). Publication in Wellcome Open Research does not imply endorsement by Wellcome.

## Introduction

The facts of the case, though they were initially met with incredulity, are now more or less established: Dr He Jiankui, a biotechnology entrepreneur and researcher at the Southern University of Science and Technology in Shenzhen, China, recruited a number of couples to a project that led to the birth, in 2018, of the first babies to have had their DNA deliberately modified using the genome editing tool CRISPR-Cas9 ^1^. The objective of these procedures was not connected with the avoidance of an imminent risk to the future children but to confer to them the advantage of inherent resistance to HIV, with which the male partner of each couple was infected. These treatments, described at the Second International Summit on Human Genome Editing in Hong Kong in November 2018, caused immediate consternation among scientists and the public, provoked numerous statements of condemnation from scientific and ethical bodies, and led to a number of academic, institutional and official investigations around the world, including a court case that ended with the conviction of Dr He and two associates in December 2019 for illegally practising medicine ^2^.

Of the many questions that the ‘gene-edited baby case’ provoked, among the most perplexing must be: given the already salient and near-universal opposition to clinical translation of human heritable genome editing (HHGE), how could those involved arrive at a point where they were prepared to carry out these unprecedented procedures? Furthermore, given that Dr He shared his intentions with many scientific peers, how were they able to go through with them despite their widely acknowledged proscription under relevant legal, professional and moral norms? The present paper offers a number of reflections that are relevant to these questions, building on presentations and discussions at the annual meeting of the Global Forum on Bioethics in Research, held in Singapore in November 2019. They include reflections on: the significance placed on patient interests and preferences; the perceived policy vacuum in the wake of technological developments in genome editing (in particular the rapid elaboration and diffusion of CRISPR-Cas9 techniques) and the significance of public opinion in this context; the complex motivations and incentives that bear upon ambitious biotechnology entrepreneurialism in contemporary China and elsewhere; the integrity, role and responsibilities of scientists and the international scientific community; the traction of global bioethical norms and missteps in cross-cultural understanding; and wider questions for society about the role of biomedical technologies in the collective response to societal challenges, such as the prevalence of HIV and genetic disease. In reflecting on the – so far as we know – singular case of Dr He and his collaborators our interest is not forensic but normative: our aims are to understand whether this case, though singular, should truly be seen as exceptional, to what extent the conditions that were conducive to Dr He’s interventions still prevail, and what needs to change in the light of the incident if we should wish to forestall a recurrence.

## Clinical, regulatory and societal conditions

Dr He originally announced the birth of genome edited twin girls on YouTube on 25 November 2018. Subsequently, at the Second International Summit on Human Genome Editing held in Hong Kong, he revealed that another woman was in the early stages of pregnancy with a genetically modified embryo. Dr He’s claims not only caused shock and controversy in the scientific community and the wider society, but also prompted urgent reflection on the application and regulation of emerging technologies in China and elsewhere ^3^. The actions of Dr He and his associates posed numerous ethical challenges to China and the world. At the same time, this event prompted the further development of ethical norms governing genome editing and stimulated public discussion. We review this event from three aspects, clinical, regulatory and societal, before proceeding to discuss its wider implications for Chinese and international research.

### Clinical practice

Three important features of good medical practice are that clinicians should (1) seek the informed consent of their patients before carrying out procedures, (2) fulfil a duty of care not to carry out procedures where the risks outweigh the anticipated benefits and (3) guard against conflicts of interest that could affect their clinical judgement.

Informed consent derives from the right of competent people to determine what happens to them (autonomy) and the need to protect from harm those who are not competent. Its exercise depends on conditions of information provision, the patient or research participant’s understanding of this information, and the absence of coercion. However, several pieces of evidence showed that Dr He’s project breached established principles of informed consent. Firstly, according to Dr He's documents, the program was registered at the Chinese Clinical Trial Registry on November 8, but the informed consent form called the program ‘AIDS Vaccine Research,’ so it is unclear whether participants were fully aware of the potential risks and benefits of the project ^4^. Seemingly, Dr He did not perform the obligation of information disclosure sufficiently, because he did not tell the participants that the transmission of HIV to a child can be effectively prevented by other safe measures, such as washing the infected father’s sperm before *in vitro* fertilization (a step Dr He’s team in fact performed) ^5^. Secondly, Dr He said that his team recruited these particular couples, in which the father was infected with HIV and the mother was not, by an HIV/AIDS volunteer group. The problem lies in that people in such a vulnerable position are open to being induced to participate in this project by the prospect of having healthy offspring; furthermore, it is likely that the potential benefits of genome editing were exaggerated, and the risks were underestimated or concealed. Thirdly, it is understood that infected people may be subject to significant pressure and discrimination in day-to-day life, for example, they may be discriminated against in employment and denied insurance by insurance companies. Given that, they may have sought a treatment that would allow their offspring to avoid a similar predicament. These factors call into question the voluntary nature of the consent. Last but not least, in article 3 of his informed consent form, the risk of the editing procedure missing its target is mentioned, and there are different detection methods to minimize the possibility of causing significant harm ^6^. But the project team did not properly take account of the risk of missing the target because it ‘exceeds the risk consequences of existing medical science and technology’, this article breached the an acceptable balance of benefits against risks for the participants ^5^. To sum up, it appears that the principles of informed consent had been violated. The problem does not end there, however: patient consent is only one element, perhaps the last element, in securing ethical treatment; prior to that are questions of science and of human values, which few would allow that individual interest should override. 

Dr He said that his aim of this research was to aid these vulnerable people, but the data he presented suggested that his experiments failed. Neither Lulu nor Nana, the twins born following the first procedure to result in a live birth, possessed the 32-base pair deletion desired in the *CCR5* gene, the gene for a protein on immune cells that HIV uses to infect the cells. Furthermore, the procedure also resulted in ‘off-target’ changes elsewhere in the girls’ genomes ^7^. Even though the risks far outweighed the benefits, Dr He carried out the procedure anyway. Consequently, Dr He’s ‘gene surgery’ posed a number of foreseeable risks to the babies and their offspring, without providing any obvious medical benefit.

Another problem is conflicts of interest. With governmental, domestic and international investment, Dr He became the owner of six companies and a significant shareholder of seven companies, with personal wealth of several billion yuan ^8^. Two of the companies in which Dr He is a shareholder are involved in genetic testing. Nevertheless, Dr He and his team declared ‘no competing financial interests’ in a paper on germline genome editing published in *The Crispr Journal* on November 28, 2019 (since retracted). Furthermore, Dr He concealed his interest in these companies when he recruited couples to participate his program. This tarnished the scientific integrity of his study and casts suspicion over his motivation.

### Regulatory policy

The authorities in Guangdong province and Shenzhen (part of Guangdong) launched a joint investigation. As reported by Xinhua, China’s state-run press agency on January 21, 2019, they found that

‘Dr He secretly organized a project team including foreign personnel, from June 2016, deliberately evaded supervision, used techniques with unclear safety and effectiveness, and carried out genome editing in human embryos for reproductive purposes, which were banned in China. From March 2017 to November 2018, Jiankui He recruited eight couples to participate in the experiment by forging ethical review papers. Because the regulations of assisted reproduction prohibit people living with HIV to use assisted reproductive technology, Dr He’s team organized other health people to take blood tests instead of these couples, and ordered some individual practitioners to conduct genome editing on human embryos and implant them into the mother's body.’ ^9^

This investigation was followed by formal legal proceedings which resulted in a judgment that was delivered on December 30, 2019 at the Nanshan District People's Court in Shenzhen. Jiankui He, Renli Zhang and Jinzhou Qin were respectively investigated for criminal liabilities for illegally carrying out human genome editing and reproductive medical activities for the purpose of reproduction, which constituted the crime of illegal medical practice. The court held that the three defendants had not obtained the necessary medical practice qualification, and had intentionally violated the state regulations on scientific research and medical management, violated the principles of scientific research and medical ethics, and rashly applied gene editing technology to human assisted reproductive medical treatment. Jiankui He was sentenced to three years in prison and fined CNY 3 million. Renli Zhang was sentenced to two years in prison and fined CNY 1 million, and Jinzhou Qin was given 18 months in prison with a two-year reprieve, and a CNY 500,000 fine ^10^.

At the time the treatments were carried out, China had not established explicit and legally binding rules or standards for interventions in the human genome. Relevant provisions are only found in the ‘Key Points for Quality Control of Clinical Research on Human Cell Therapy and Gene Therapy’ published by the Former Ministry of Health in 1993, and the ‘Interim Measures for Human Genetic Resources Management’ published by the State Council in 1998. However, the (non-binding) 2003 ‘Ethical Guiding Principles for the Research of Embryonic Stem Cell’ issued by China’s Ministry of Science and Technology and the Former Ministry of Health (now National Health Commission) unequivocally rule out any research beyond 14 days after the creation of an embryo as well as any implantation of a genetically modified embryo into the human reproductive system ^11^. The activities of Dr He’s team led to the birth of two baby girls and a further pregnancy, which breached these principles. In addition, it was found that ethical review materials had been forged by Dr He’s team to evade supervision, the existence of informed consent was questionable, and the project violated the Biomedical Research Ethics Review Method Involving People published by the National Health Commission ^12^.

### Societal consideration

Public opinion on this event is mixed. In 2019, the Key Laboratory of Public Opinion in Big Data Analysis and Simulation of Guangdong Province at Sun Yat-Sen University released the first research report on the public's understanding and attitude towards gene editing technology in China ^13^. They report the results of a survey of the attitudes of the public and people living with HIV towards genome editing techniques. The report shows that more than 60% of the Chinese public supported therapeutic use of genome editing in adults and children, while nearly 70% of people reject the use of genome editing for conditions that are not genetic or for non-medical purposes, such as to enhance IQ or athletic abilities. Over 60% of people believed that the Government should provide more funding for genome editing ^14^. Such a supportive attitude favors the promotion of research in genetics and also demands rigorous ethical governance and oversight of emerging technologies. Ensuring the healthy, ethically warranted, sustainable development of science is always of paramount importance for the well-being of humankind.

Dealing with the relationship between scientific advance and the development of public policies presents challenges. Scientists will always endeavor to make breakthroughs, while policy makers strive to secure that the use of technology benefits people safely. However, the development of policies can often seem to fail to keep pace with developments in technology and, while the publics’ voice is usually ignored in scientific areas, events such as the ‘gene-edited baby’ case expose the gap between them. Some people assert the importance of attending to public opinion on genome editing, because this can offer a new perspective; more importantly, as they will also be affected by genome editing, they have a presumptive right to know about new developments and take part in the governance of scientific research. Besides, the impact of emerging technologies is so widespread, there are issues with new technologies that cannot be left to clinicians and patients but which are properly the domain of public policy, and, arguably, international ethical debate. 

## Political, cultural and economic conditions

Then, why China? From a broad political, sociological and historical perspective, Dr He’s genetic transgression is not an isolated case. Set within the context of China’s approach to biomedicine and bioethics and its global ambitions, Dr He’s experiments fit into a radical and dangerous climate. It is true that his action was condemned by the genome editing community in China and other committees and government agencies, such as the Genetics Society of China, the Chinese Society for Stem Cell Research ^15^, and the National Health Commission in China ^16^, rightly pointed out that Dr He seriously violated ethics, scientific research integrity and relevant state regulations, causing adverse effects at home and abroad. However, as Nie rightly pointed out, to blame Dr He alone disregards the wider responsibilities of his university and other authorities ^17^. In the past several decades, a permissive regulatory climate and a pragmatic approach have fostered unprecedented growth of the biomedical revolution in China. This climate has created a fertile environment for Chinese researchers to pursue daring but unethical ‘world firsts’. Furthermore, as there are also structural, systematic and institutional flaws behind this experiment, it is important to examine ‘what created him, what incentivized him and what failed to stop him’ ^18^.

### Development of biomedicine in China: strategic national goal

The Chinese government recognized that bioscience could play a major role in its global competitiveness and determined to be a key player and leader. Biomedicine, synthetic biology, brain research and regenerative medical techniques are listed as strategic fields and industries in China’s ‘13th Five-Year Plan’ National Strategic Emerging Industry Development Plan and Healthy China 2030 strategy ^19^. Life science and health-related projects account for 17 out of 60 targeted areas with high investment priority. In the ‘13th Five Year Plan’, ‘The Program of Six New Free Trade Pilot Zones’ and other programmatic documents related to economic and social development all have incentive policies for industrial development in the field of gene therapy. In terms of technology and industry promotion, gene therapy has wide ranging prospects both as a new medical technology and as a biomedical industry. As China’s healthcare industry grows and diversifies, so do the opportunities – its healthcare market is expected to reach CNY 198 billion (USD 28.59 billion) in 2026, increasing tenfold from 2016 ^20^. China has also become the top destination for research involving primates, which are invaluable models for studying human disease, especially in the brain. Other countries do not breed primates in such large numbers or to the standard produced in China. In research that is controversial internationally but which drew little attention domestically, researchers from the Chinese Academy of Sciences have genetically modified monkeys so they show autistic behaviors with the aim of increasing understanding of brain disorders ^21^. Another controversial case was that scientists from Shanghai have created the first primates cloned with a technique similar to the one used to clone Dolly the sheep. Researchers hope to develop populations of genetically identical primates to provide improved animal models of human disorders, such as cancer and Parkinson’s disease ^22^.

In the Nature Index, China is the second leading contributor to biomedical engineering articles after the United States, measured by its contribution to the authorship of papers in 82 high-quality research journals in 2015–17 ^23^. With respect to CRISPR, in a recent analysis of more than 2,000 patent applications for distinct inventions that involved CRISPR, the United States barely edged out China: publicly available CRISPR patent applications as of December 2018 show that the United Sates has 872, China 858 and Europe 186. Applications from China have climbed rapidly in recent years, and the country dominates in the agricultural and industrial realms.

### World’s first: Catching up with – and surpassing – the West with translation

The genome editing experiment was not the first time Jiankui He had gained publicity and attention in China. Earlier in 2017, Chinese state broadcaster CCTV showed a series promoting China’s achievements in science and technology ^18^. One episode profiled a Chinese scientist who claimed to have invented a gene-sequencing machine that outperformed those in the West. ‘Somebody said we shocked the world with our machine’, Dr He said in front of the camera with a proud smile ^18^. Another interesting phenomenon was that when Dr He’s research was first reported, in November 2018, the *People’s Daily Online*, one of the most influential official media outlets, promoted and celebrated it as ‘the world’s first gene-edited babies genetically resistant to AIDS were born in China’, and ‘a historical breakthrough in the application of gene editing technology for disease prevention’ ^24^. However, as more detailed information about his work was released and also the worldwide outcry and condemnation was reported by media, the *People’s Daily* quickly deleted that news item.

He Jiankui used rhetoric such as ‘world’s first’ and ‘surpassing the West’, which are sensational, nationalistic, and stimulating. This rhetoric also resonates with the grander Chinese dream, chased by the government and society, of catching up and surpassing the West. Dr He received extremely generous support from central and local governments and scientific organizations. More importantly, he was selected for the Central Government’s top science program, the ‘Thousand Talents Plan’. With governmental and domestic investment, he has become the owner or significant shareholder of at least seven genetic biotechnology companies ^17^. Perhaps, when he announced the ‘world’s first’ genome edited babies, he was expecting the congratulations and acclaim that he had always been accustomed to in China. To some extent, Dr He’s human experimentation constitutes one of the fruits of his personal ambition ‘nourished and supported by China’s drive for superpower status in science, technology, and medicine.’ ^17^

In recent decades, the Chinese government has strongly promoted the transformation of scientific discoveries and technological inventions into clinical practices, products and economic growth. In 2015, the Communist Party of China Central Committee and the State Council issued a policy document on ‘strategy for innovation-driven development’. In August 2016, the Ministry of Science and Technology and the Ministry of Education issued ‘Opinions on Strengthening the Role of Higher Education Institutions in Transfer and Transformation of Scientific and Technological Achievements.’ ^25^ University researchers, including students, are encouraged to transform their scientific investigations and technological inventions into economic development. This also becomes one of the evaluation standards for universities, which are required to submit their performance on translating scientific research into economic growth and production annually. These policies aimed to promote scientific research and innovation but, in reality, scientists and medical doctors receive conflicting financial incentives and face serious ethical issues in the process of translating their research into industrial processes or products. At the same time, China still lacks rigorous ethics governance and oversight, especially on conflict of interest. As Nie has rightly pointed out:

‘as manifested in the rising cases breaching scientific integrity and especially He Jiankui’s human experiments, China’s science schemes have much to do with the developing mentality that ethics is merely secondary and instrumental for cutting-edge scientific investigation and technological invention. Ethical considerations and the ultimate moral goals of science and medicine can be compromised or alienated by the unchecked pursuit of personal ambition, financial interests, interests of the party-governments and institutions, economic growth, or national glory.’ ^26^

A similar example is the growing business of stem cell tourism in China: although many concerns have been raised regarding fraudsters that operate unsafe stem cell therapies, the local officialdom ‘turns a blind eye to the questionable technology.’ ^27^ Increasing government funding of scientific research has promoted rapid developments in stem-cell research in China. The number of translation studies, including basic and preclinical investigations, has also increased. Around 100 stem-cell banks have been established in China, 10 stem-cell drugs are currently in the approval process, and more than 400 stem cell-based clinical trials are currently registered in China ^28^. The Chinese regulatory approach to biomedical research is based on Guidelines and Administrative Measures, rather than legislation. Therefore, the force of governance measures is very limited, and certain institutions such as military hospitals are not subject to even this level of control. In 2016, Wei Zexi, a 21-year-old student died after receiving an experimental immunotherapy cancer treatment at the No 2 Hospital of the Beijing Armed Police Corps ^29^. Wei underwent the procedure, which cost his family more than CNY 200,000 (USD 29,130), after using online search engine Baidu to research treatments. The hospital’s details topped the list returned by Baidu, but it failed to save Wei.

After the ‘Wei Zexi incident’ involving biological immunotherapy in 2016, the National Health and Family Planning Commission immediately suspended all unapproved clinical applications of the third class of medical technology (those designated by the Ministry of Health as having significant ethical issues and higher risks, whose safety and effectiveness are yet to be proved), and the regulatory policies on gene therapy were revised ^24^. Under the new policies, the admission and management of the second‑ and third‑class technologies was moved from health administration departments to the medical institutions providing the treatment to ensure that process could be more easily tracked ^30^. This approach has already been adopted by many developed countries such as France, Britain, and Canada.

### Lack of compelling ethical oversight and regulation, difficult implementation

Although many hospitals and universities in China have established institutional review boards (IRBs) and bioethics centers in accordance with internationally recognized principles, they are not home-grown institutions ^31^. For many researchers, they are treated as imported Western bureaucratic instruments not rooted in Chinese culture. In the West, the Nazi medical experiments in Europe, the Tuskegee syphilis experiment in the US and other scandals led to the establishing of IRBs, which approve and oversee and supervise medical experiments involving human subjects. In China, there is also a history of notorious human experiment including the Japanese army unit 731 that conducted experiments on Chinese citizens during World War II, but it did not give rise to the development of practices and institutions of research ethics as in the West ^31^. Some studies have revealed IRBs are not properly established and consistent in practice, mainly owing to their limited experience in handing relevant ethical questions, or coming under pressure from the authorities, or sometimes existing merely for the sake of formality ^32, 33^.

Despite ethical guiding principles and management measures applied to research involving human embryonic stem cell and human assisted reproductive technology, up till now China has not formed a comprehensive and systematic, legally underpinned regulatory system for human genetic technology issues, including genome editing and gene therapy. The current oversight norms are mainly technical management methods and ethics principles at a low-level, with no directly applicable legislative provisions. In contrast to US and European regulations, they are not enforceable by law. In response to the ‘gene-edited baby’ case, the Chinese government brought forward legislation in areas such as biosecurity, genetic technology, and biomedicine. In early 2019 the National Health Commission issued draft regulations on the clinical applications of the new biomedical technologies, focusing on so-called ‘high-risk’ biomedical technologies like human germline genome editing, which need to be reviewed at national level ^34^. In July 2019, China approved a plan to establish a national commission for ethics in science and technology. The National Science and Technology Ethics Committee is charged with promoting the development of ‘a more comprehensive, ordered and coordinated governance system’ in 2020 ^35^; meanwhile, the ‘Biosafety Law’ has passed the second review by the Standing Committee of the National People’s Congress and is currently under public consultation ^36^.

## Silence and complicity

Beyond the Chinese context, there was a substantial number of individuals around the world whom He Jiankui had informed about his experiment prior to its public release. These individuals have been referred to as part of Dr He’s ‘circle of trust’ ^37^. The extent to which they were aware of the trials varied, as well as their reactions to the information. None, though, decided to release the information of the first reproductive use of gene editing to the broader scientific community or public at large.

There is reason to believe that releasing information earlier could have prevented the experiment from proceeding with the pregnancy that resulted in the first live births of twins, or if made after the first live births, that it could have prevented the pregnancy that resulted in the third live birth that was recently confirmed by a Chinese court ^38^.

### Silence

The whistleblowing literature distinguishes between ‘internal whistleblowing’ – involving organizational structures and processes- and ‘external whistle blowing’ – involving external bodies such as ‘agencies, professional bodies, regulators and the media’ ^39^. In almost all cases of whistleblowing, concerns are raised internally on more than one occasion before the whistleblower turns to external bodies ( *ibid*). The case of He Jiankui is complicated by the fact that the ‘circle of trust’ comprised individuals external to Dr He’s organization, who, further complicating the issue, were mostly based abroad. Given this, releasing information about Dr He’s experiment would not strictly have been a standard case of whistleblowing. However, theoretical and terminological issues aside, the central question that remains is: What should we make of the failure to release information by those who were in a position to do so, ethically speaking?

Clearly, it was a case which should have raised ethical concerns for the ‘circle of trust’ as evidenced by the world’s reaction to it. Dr He’s experiment was widely derided as premature due to scientific and safety concerns. The ‘circle of trust’ must have known that this was an ethically dubious experiment that required public deliberation considering the numerous statements on this issue. For example, a widely-publicized and discussed statement from the organizing committee of the 2015 International Summit on Human Gene Editing stated that ‘It would be irresponsible to proceed with any clinical use of germline editing unless and until (i) the relevant safety and efficacy issues have been resolved, based on appropriate understanding and balancing of risks, potential benefits, and alternatives, and (ii) there is broad societal consensus about the appropriateness of the proposed application.’ ^40^ Indeed, Dr He himself wrote that proceeding with germline editing would be ‘extremely irresponsible’ without resolving safety issues – just weeks before commencing his experiment ^37^.

Given the substantial benefit that would have resulted from stopping the experiment, one might be led to believe that there was a duty to release such information. Initial support in favor of sounding the alarm about the experiments might come from the general ‘duty of easy rescue’, a plausible moral principle stating that if one could bring about a substantial benefit or prevent substantial harms to others at little cost to oneself, one should do so. However, this principle does not apply in the current case because the potential reputational cost of releasing such information could be substantial. These individuals would likely have suffered reputational harm, as others may have deemed them as ‘uncooperative troublemakers’ and become unwilling to engage in scientific collaborations or communication with them. Furthermore, scientific correspondence such as that sent out by He Jiankui is typically made under a presumption of confidence; therefore, reporting such correspondence may have been seen as a breach of trust.

As has been argued elsewhere ^41^, a more promising way of grounding the duty to release information may be found in a scientist’s professional responsibilities as they are articulated in recognized professional codes of conduct. According to these codes, scientists have a responsibility to report suspicions of wrongdoing and research misconduct, on the grounds that such instances ‘undermine the trustworthiness of research’ ^42^. Unlike the ‘duty of easy rescue’, the professional responsibility to report instances of research misconduct, including ethical failures, would hold even if there are substantial reputational costs involved for individuals who release the information.

### Complicity in the experiment

A further point worth exploring is the idea that the members of Dr He’s ‘circle of trust’ were, to an extent, complicit in the unethical experiment due to their silence. This can be best understood by applying Chiara Lepore and Robert Goodin’s account of ‘complicity’, which holds that performing an action (or, in some cases, an inaction, as described below) ‘that contributes to the wrongdoing of another and knowing that it does so (but without necessarily sharing the other’s wrongful purpose), […] are minimally required for one to be complicit with the wrongdoing of another.’ ^39^ For someone to be complicit in wrongdoing means that that person shares at least some of the blame for the wrongdoing. If those in the ‘circle of trust’ were complicit, they would not only be criticizable for failing to discharge a duty to report wrongdoing, but could furthermore be deemed partly responsible for the wrongdoing itself. This raises the moral stakes, so to speak, of failing to report, and in turn provides additional motivation to support reforms that ensure scientists report future wrongdoing.

As described above, there were numerous aspects of wrongdoing in He Jiankui’s experiment. Some details, including inadequate consent, improper medical procedures, and alleged unverified approvals, would not have been accessible to individuals with whom He Jiankui was in communication. Nevertheless, they were reportedly informed about the more basic facts of the case, that He Jiankui was editing the DNA of embryos to prevent HIV transmission, and implanting those embryos ^37^. They also would have or should have known that such a procedure was premature due to concerns about efficacy and safety of the intervention as well as a lack of social license. Pronouncements of well-established national and international bodies over several years had been unanimous on that front ^43^. The basic facts on their own would be sufficient to merit condemnation of the study and necessitate its closure.

Notably, most members of the ‘circle of trust’ were not actively involved with any study procedures – they were just kept informed. Because complicity with wrongdoing implies a degree of blameworthiness, and blame is only appropriate when there is some sort of causal contribution, complicity in turn requires some sort of causal contribution. One might argue that omissions cannot be causes, and so in this case members of the ‘circle of trust’ were not complicit. We, however, find that Lepore and Goodin’s rejoinder relating to omission is helpful: ‘What is crucial in making something a causal contribution is the fact that had you done something else, the wrongdoing would not have occurred.’ ^44^ This counterfactual is almost certainly fulfilled in the present case, as the eventual public release of information did lead to the experiment’s immediate closure.

The case for scientists’ inaction being a causal contributor is bolstered by their duty to release information about deeply unethical research. For such cases, Lepore and Goodin tell us that ‘[w]here there is a duty to do something and you do nothing, your ‘doing nothing’ counts as a cause.’ ^39^ Thus a life guard who does not take any action to save a child from drowning in a swimming pool would be deemed responsible for contributing casually to the child’s death precisely because the life guard was under a duty to watch over the children ^39^. Scientists may not be in exactly the same role as a lifeguard, but, as argued above, scientists may nevertheless have professional responsibilities in relation to wrongdoing of their peers.

Still, we should not overstate this claim. Complicity comes in degrees, attenuated by the centrality of causal contribution. Moreover, the extent to which complicit individuals are blameworthy depends on the ‘extent of contribution, and extent of shared purpose with [a] principal wrongdoer.’ ^45^ In this case, most members of the circle of trust were not integrally involved in the study design and conduct, so could hardly be considered central players. As for endorsement, some apparently responded positively to Dr He when informed of the experiment, others negatively ^37^. A charitable interpretation, based on subsequent statements, is that generally members of the ‘circle of trust’ did not strongly endorse the study’s aims and therefore did not share Dr He’s purpose.

Beyond the individuals involved, a question can be raised about the role of the broader scientific community. A culture in which those who inform on unethical behavior of colleagues are ostracized and disregarded, and conversely risk-takers are rewarded for even questionable experiments as long as the outcomes are successful, foreseeably contributes overarchingly to wrongdoings of the kind that He Jiankui committed ^45^.

Because the scientific community was not generally informed of He Jiankui’s experiment, this community cannot strictly be said to be complicit in the wrongdoing. Nevertheless, we should keep squarely in view the effects of pervasive scientific culture. This culture might enable or tacitly encourage not only He Jiankui’s experiment itself but also the decision by those in the ‘circle of trust’ not to inform the scientific community, or society more broadly. Preventing future wrongdoing will require critically reflecting on the factors, including scientific culture, that possibly contributed to the ‘circle of trust’ becoming complicit.

### Taking responsibility

We have, then, a degree of complicity in He Jiankui’s experiment by members of the ‘circle of trust’, and the contributory role of the international scientific community towards that complicity. This highlights the extent to which individuals and groups that need to take responsibility for their actions – and inactions – in order to improve practice going forward. In particular, work is needed to remove the barriers that prevent reporting of instances of wrongdoing and thereby facilitate complicity.

Elsewhere, it has been argued that one concrete step to improve the likelihood of scientists coming forward would be to create an international reporting mechanism. This would at least partially address the practical problem of there being no obvious body to report to when gene editing research occurs abroad ^41^.

Another complementary approach is to push back against the norm of silence. In this case, there may have been a presumption of confidentiality on the part of He Jiankui, that those to whom he sent communications would not leak or otherwise disclose the contents. In general, such a presumption may be well-justified. Beyond general privacy concerns, making private scientific communications public might have a chilling effect on scientific discourse, discouraging honest discussion that might be misinterpreted if released without appropriate context ^46^. It could also discourage innovation by allowing third parties to ‘scoop’ certain novel approaches before they are ready to be implemented.

However, it would be inappropriate to infer from this an absolute right of confidence. In the area of moral rights, the right of confidence is merely *prima facie*: a relevant moral consideration that can be overridden by competing considerations. As for legal rights, there is no general legal right to confidentiality in scientific communications analogous, for instance, to physician-patient or reporter-informant confidentiality ^47^.

The *prima facie* right to confidence would presumably not preclude the release of information that is unambiguous evidence of research fraud, even if this information emerged from personal correspondence. Likewise, clinical research involving human heritable genome editing in the present state of scientific research and public discourse would be just the sort of consideration weighty enough to outweigh any such *prima facie* right.

It is a much more complex and difficult task to identify and evaluate practical actions that would be effective at reforming those norms of silence – too ambitious for the scope of the present discussion. We will simply note, though, that the scientific community should take active steps to work towards such reforms, not just in the area of germline gene editing, but for the wider array of scientific conduct. The silence and complicity in the case of the first gene-edited babies is not an isolated incident, but part of a broader pattern of scientific behavior around the world that calls out for reform.

## Conclusion

It would be convenient to cast He Jiankui as rogue or maverick, the bearer of sole responsibility, working independently of the wider research community, institutions and systems. Whatever the details of the case itself, however, this cause célèbre has drawn attention to a number of contributory conditions, the persistence of which cannot and should not be ignored. These include the way in which biomedical innovations are implicated in multiple systems of practice (those of research, medicine, business, techno-nationalism and responses to broader societal challenges) and rely on the support or complicity of others (of colleagues, patients, mentors, institutions and officials).

On the question of how this project could come about in the first place, given the ostensibly overwhelming opposition to clinical translation of HHGE, we risk ignoring powerful contributory conditions if we treat biomedical technologies as simply the outworkings of scientific research. As we have shown in this paper, the ‘gene-edited baby’ case reveals the extent to which the traction of other interests, such as those of personal ambition, national prestige and economic reward can intervene in biomedicine. But no more can biomedical technologies be thought of as simply a response to clinical need. Though *CCR5* had been an early target for proof-of-concept research, few can have expected the first efforts to edit the human genome to have aim of conferring resistance to an easily avoidable disease. To make such assumptions is, however, to ignore the local conditions of access to assisted conception treatment for those with HIV, the societal challenge it represents and the waiting market for expanded reproductive choice. When the object was to pioneer a new service, the choice of target that might have seemed achievable and publicly popular, rather than one that was clinically desirable (such as obligatorily heritable disease), could have appeared perfectly reasonable: cracking *CCR5* would demonstrate a translational pathway for other indications to follow ^48^. This becomes more plausible if HHGE is understood not as a therapeutic intervention but as a technology for expanding reproductive options ^49^. But this dichotomy, too, is merely complacent – it suggests that evaluation can be laid out on a simple scale, though in reality it is much more complicated.

On the question of why a vaulting ambition like that of He Jiankui could not be brought down to Earth, despite the fact that it was shared with many eminent and influential scientists, once again, it is easy to defer to failures of formal oversight or the absence of clear and effective sanctions. But while relevant instruments and procedures existed to an extent, the prevailing culture flowed around them and built networks of circumvention. As we have shown in this paper, a culture of responsible research cannot rely on individual actors calculating what is in their own best interests. The confusion of entrepreneurialism with science only makes the gaming of responsibility more likely. The response of those who knew of Dr He’s intentions was an example of a phenomenon that explains failures of collective action in fields from the prevention of anthropogenic climate change to financial crises, that of ‘organized irresponsibility’, where responsibility is diffused and passed around a system rather than vested in any identifiable actor ^50^. Failures by omission can be seen as non-culpable because it can be assumed that either someone else will see to it or, if not, all will be equally culpable (and therefore none will be). 

We cannot say that the ‘gene-edited baby’ case has been salutary, so long as many of the conditions that nourished it remain. The prevention of future events of this kind must begin with an understanding of these conditions rather than the search for causes and the assigning of liabilities. 

## Data availability

### Underlying data

No data are associated with this article.
