# Supplementary material for: Making sense of it all: Ethical reflections on the conditions surrounding the first genome-edited babies
Source: Wellcome Open Res. 2021 Jun 23;5:216. Originally published 2020 Sep 16. [Version 2] doi: 10.12688/wellcomeopenres.16295.2 (PMC8340653; doi:10.12688/wellcomeopenres.16295.2)
Supplement: Peer review history

# Peer review: Reviewer response for version 2

**DOI:** 10.21956/wellcomeopenres.18740.r44615

**Published:** 2021-08-04

**Authors:** Giulia Cavaliere

**Affiliations:** 1 Dickson Poon School of Law, King's College London, London, UK

Thank you for taking the time to read our reports and engage with our comments. The revised version is excellent, and I take your point about breadth. There's certainly a value in that, especially when the paper is the product of a number of authors with such a heterogeneous expertise.

Does the article adequately reference differing views and opinions?

Partly

Are all factual statements correct, and are statements and arguments made adequately supported by citations?

Partly

Is the Open Letter written in accessible language?

Yes

Where applicable, are recommendations and next steps explained clearly for others to follow?

Yes

Is the rationale for the Open Letter provided in sufficient detail?

Yes

Reviewer Expertise:

Applied Ethics, Political Philosophy

I confirm that I have read this submission and believe that I have an appropriate level of expertise to confirm that it is of an acceptable scientific standard.

# Peer review: Reviewer response for version 2

**DOI:** 10.21956/wellcomeopenres.18740.r44616

**Published:** 2021-07-15

**Authors:** Yann Joly

**Affiliations:** 1 Center for Genomics and Policy (CGP), McGill University, Montreal, Canada

I agree with the changes and justification provided by the authors. I only have one minor point of contention left.

 Regarding the statement of the author that:

 "The paper tries to show how the political and scientific climate and pragmatic approach have fostered unprecedented growth of the biomedical revolution in China. This climate has created a fertile environment for Chinese researchers to pursue ‘world firsts’. Some Chinese media and press industry are ignorant of bioethical principles and values (both Chinese and other values), but that does not mean Chinese academic community (both scientific and bioethics) and medical authorities are indifferent or ignorant of the medical ethics principles. On the contrary, many Chinese academic bodies issued statements condemning the experiment quickly. Also, from personal communication with many scholars, they feel ashamed that Chinese media did not respond to this event appropriately."

 I agree with the authors that many Chinese scholars in this field, including He Jiankui, are well aware of the core bioethics principles. However, knowledge of the norms might be in itself insufficient. Researchers need to have integrated their content in their practice to a point where they've become part of the scientific & social norms of the field.  In China, the interest in bioethics and adoption of such norms are a recent event. Furthermore, there remains important uncertainties regarding how several of these norms should be applied to concrete cases. Together this context explains why some Chinese scientists, while well aware of the law & policies, still decide to attempts "world firsts'' that are unethical.

Does the article adequately reference differing views and opinions?

Partly

Are all factual statements correct, and are statements and arguments made adequately supported by citations?

Yes

Is the Open Letter written in accessible language?

Yes

Where applicable, are recommendations and next steps explained clearly for others to follow?

Yes

Is the rationale for the Open Letter provided in sufficient detail?

Yes

Reviewer Expertise:

NA

I confirm that I have read this submission and believe that I have an appropriate level of expertise to confirm that it is of an acceptable scientific standard.

# Peer review: Reviewer response for version 1

**DOI:** 10.21956/wellcomeopenres.17906.r42270

**Published:** 2021-02-09

**Authors:** Yann Joly, Song Lingqiao

**Affiliations:** 1 McGill University, Montreal, Canada; 2 Center for Genomics and Policy (CGP), McGill University, Montreal, Canada

This very well written, ambitious, manuscript seeks to provide an account of the circumstances that led to He Jiankui’s controversial germline gene editing experiment that resulted in the birth of genetically edited twins. While it does a good job as an open letter, the broad scope of factors (political, legal, cultural and economic) considered, prevents a deep treatment of each of these circumstances. The result is an interesting attempt to explain the circumstances of the gene editing incident and to identify changes needed to prevent similar ones to happen again in the future. However, given the summary treatment given to different factors identified, this account should be accepted with some reserve. Claims will need to be carefully assessed and validated through additional in-depth research.  

 In  the section of ‘political, cultural and economic conditions’, the authors could write more about the Chinese cultural differences with Western countries. For instance, Confucianism influenced the Chinese public perception of human life through its various stages of development and of the concept of humanness. This deeply rooted tradition likely contributed to a full account of He Jiankui’s incident.

 On page 4, I the authors claim that ‘‘China had not established explicit and legally binding rules or standards for interventions in the human genome’’ needs additional nuances. Technical guiding standards published by the Ministry of Health were in place, but the issue is these documents have low binding power.

 In addition, on page 4, one important document was missing for the section, which is Article 22 of the Management Statement on the Human Assisted Reproductive Technology (人类辅助生殖技术管理办法). For a complete list of related laws and regulations on gene editing, you can refer to this book chapter.

 On page 5, the authors mentioned at the very beginning, that He Jiankui was praised by the Chinese media. This fact also reveals a lack of familiarity with western bioethics principles and values within the Chinese press industry and other professional groups, as well as, the general public. This is also an important reason why He Jiankui’s case occurred in China.

 On page 7, the authors mentioned several regulatory reforms on gene editing. But this enumeration is incomplete. More relevant regulations have been released in response to He Jiankui’s incident. I suggest updating the regulatory paragraph accordingly.

 Additional references are needed in some places to support the claims of the authors: For instance, on page 3, ‘They include reflections on: the significance placed on…genetic diseases’ ‘Three important features of good medical practices…’ Also, on the same page, ‘Informed consent derives from …of coercion.’ On page 6, ‘This approach has already …’ On page 5, ‘some people assert …ethical debate.’

Does the article adequately reference differing views and opinions?

Partly

Are all factual statements correct, and are statements and arguments made adequately supported by citations?

Yes

Is the Open Letter written in accessible language?

Yes

Where applicable, are recommendations and next steps explained clearly for others to follow?

Yes

Is the rationale for the Open Letter provided in sufficient detail?

Yes

Reviewer Expertise:

East-Asian Bioethics & Policy Studies, Genetic Tests, Identity and Equity, Health Law, Intellectual Property Law, International Human Rights Law, Social Sciences Research Methods

We confirm that we have read this submission and believe that we have an appropriate level of expertise to confirm that it is of an acceptable scientific standard, however we have significant reservations, as outlined above.

# Response

**Published:** 2021-06-08

**Authors:** Peter Mills

**Affiliations:** Nuffield Council on Bioethics, UK

We are grateful to both reviewers for the trouble they have taken to offer their thoughtful and helpful comments.

 Both reviews note the difficulty with the ambition to cover such a range and variety of issues. This arises from three considerations. The first is its genesis in presentations at the Global Forum on Bioethics and Research in 2019, the virtue of which was to bring together complementary perspectives that could be mutually illuminating and together foster a more complete understanding of the subject. The second is, as both reviewers note, that there is an extent to which there must be a trade-off between the virtues of breadth and depth, and the aim in the present paper was to take advantage of the multiple authors’ perspectives to pursue the first virtue while in no way foreclosing the second. The third is the selection of the Open Letter format, as an appropriate vehicle for this, in that it offers scope to identify and juxtapose many of the key points of interest, which, as the second reviewer implies, in effect defines a programme of further research, contextualises it and signposts how it may be pursued. We are, nevertheless, conscious that some of the signposts might be missing and others could be clearer, which we have endeavoured to address through additional referencing and clarifications in the text.

**In response to specific comments:**

 Regarding the section on ‘political, cultural and economic conditions’, we acknowledge that Confucianism is an influential ethical theory in China and presents a distinctive understanding of human life, dignity, humanness, etc. Confucian understanding of human life and dignity would contribute a valuable perspective to the question of whether the application of gene editing technology related to human birth violate human dignity and humanness. However, the discussion of this issue would be beyond the scope and focus of this paper, despite it is worthwhile to explore in another attempt.

 Regarding the nuance with regard to rules and standards in place relevant to He Jiankui's experiment, the reviewer is right: technical guidelines and standards were in place, but the problem is either these documents have low legal binding power, or the guidelines have no ethical components. For example, the Chinese Government’s 2003 legislation (the one explicitly prohibits genetic manipulation of human gametes, zygotes, and embryos for reproductive purposes) belongs to Departmental rule, which generally cannot be used for conviction by judges (so could not be used in the He Jiankui case). We have amended the text for clarity.

 We are grateful to the reviewers for their advice regarding effective legal documents and have added a reference to the Measures for the Administration of Human Assisted Reproductive Technology of 2001.

 Regarding He Jiankui's initial lauding by the Chinese media, the reviewer notes that this suggests a lack of familiarity with international bioethical principles and values. The paper tries to show how the political and scientific climate and pragmatic approach have fostered unprecedented growth of the biomedical revolution in China. This climate has created a fertile environment for Chinese researchers to pursue ‘world firsts’. Some Chinese media and press industry are ignorant of bioethical principles and values (both Chinese and other values), but that does not mean Chinese academic community (both scientific and bioethics) and medical authorities are indifferent or ignorant of the medical ethics principles. On the contrary, many Chinese academic bodies issued statements condemning the experiment quickly. Also, from personal communication with many scholars, they feel ashamed that Chinese media did not respond to this event appropriately.

 We have generally updated the legal references to be correct at the time of submission. Additional references have been supplied throughout.

 We remain grateful for the attention and advice of the reviewers and hope that the revisions have addressed their concerns satisfactorily.

# Peer review: Reviewer response for version 1

**DOI:** 10.21956/wellcomeopenres.17906.r40531

**Published:** 2020-09-25

**Authors:** Giulia Cavaliere

**Affiliations:** 1 Dickson Poon School of Law, King's College London, London, UK

Thank you for the opportunity to review the manuscript titled: Making sense of it all: Ethical reflections on the conditions surrounding the first genome-edited babies.

 It is an interesting article that sets out to trace and discuss key ethical, political and legal aspects of the first "'gene-edited baby' case". It is generally well written and it provides an interesting analysis of some of the key ethical issues surrounding the clinical use of CRISPR-Cas9 by He Jiankui. It lacks some depth of analysis due to the variety of issues that it discusses, but it is overall an important contribution to the literature on the ethics and governance of emerging technologies (genetic and non). In my view, the article would benefit from being revised according to some of the comments that I have outlined below.

- One of the strengths of this paper is the breadth of the analysis that it carries out. It touches upon many significant issues, from informed consent and complicity to broader considerations concerning science culture of silence and societal views on emerging technologies. Such breadth however also represents one of the weaknesses of the paper, in that some reflections are significantly underdeveloped and do not engage with the existing literature on the ethics and governance of gene editing. Some examples:
  - The section titled 'Societal Considerations' is vague and underdeveloped. It starts with reporting the results of a survey, then it moves to a brief mention of 'ethical governance' and it incorporates some sweeping claims such as 'science will always endeavor to make breakthroughs, while policy makers strive to secure that the use of the technology benefits people safely". While this is broadly correct, it represents a crude generalization of the pursuit of scientific inquiry and efforts aimed at governing it. Moreover, there has been a substantial debate on these issues - none of which is referred to in this paper.
  - The second paragraph on p. 3 is symptomatic of one of the general problems of the paper, namely to try to achieve too much. As a result, some sections read a bit list-like and are just mentioned and subsequently dropped (see for instance the list that follows "they include reflections on:" in said paragraph).
  - The section on Taking Responsibility is also significantly underdeveloped and it seems to reduce questions of responsibility to confidentiality and breaches thereof. These are just some examples.
- Another worry I had whilst reading this paper concerns the value system that it implicitly refers to. One thing is saying that China falls short on having regulatory systems and governance of science in place, but it is quite another thing to say that "China lacks rigorous ethical governance and oversight". From my reading of the paper (but I might be wrong), the value-system used to appraise what HJ did (and what China did not?) is 1) western 2) universalised and 3) realist (from a meta-ethical point of view). That is: what HJ did is ethically problematic if one looks at it, as the authors do, from ethical frameworks and values that are widely shared in the West. But what is the value of carrying out such a reflection? In particular, the sections under the heading "Political, cultural and economic conditions" are quite problematic in that the authors carry out an analysis of what went wrong in China without considering the specificity of local ethical framework and governance, giving the impression of treating China as the "far east" so to speak.
- Relatedly, and as the authors mention later on in the paper, most of the people who did not speak out against HJ were Western scientists. So why then insisting that the problem is necessarily "local", i.e. the product of a lax approach to ethics and governance?
- Lastly, it might be that it is appropriate for this specific format (open letter) not to reference other works on the issues discussed in the paper, but given the wealth of scholarship produced in the last two years, it is puzzling to see so few references in this paper.

Does the article adequately reference differing views and opinions?

Partly

Are all factual statements correct, and are statements and arguments made adequately supported by citations?

Partly

Is the Open Letter written in accessible language?

Yes

Where applicable, are recommendations and next steps explained clearly for others to follow?

Yes

Is the rationale for the Open Letter provided in sufficient detail?

Yes

Reviewer Expertise:

Applied Ethics, Political Philosophy

I confirm that I have read this submission and believe that I have an appropriate level of expertise to confirm that it is of an acceptable scientific standard, however I have significant reservations, as outlined above.

# Response

**Published:** 2021-06-08

**Authors:** Peter Mills

**Affiliations:** Nuffield Council on Bioethics, UK

We are grateful to both reviewers for the trouble they have taken to offer their thoughtful and helpful comments.

 Both reviewers note the difficulty with the ambition to cover such a range and variety of issues. This arises from three considerations. The first is its genesis in presentations at the Global Forum on Bioethics and Research in 2019, the virtue of which was to bring together complementary perspectives that could be mutually illuminating and together foster a more complete understanding of the subject. The second is, as both reviewers note, that there is an extent to which there must be a trade-off between the virtues of breadth and depth, and the aim in the present paper was to take advantage of the multiple authors’ perspectives to pursue the first virtue while in no way foreclosing the second. The third is the selection of the Open Letter format, as an appropriate vehicle for this, in that it offers scope to identify and juxtapose many of the key points of interest, which, as the second reviewer implies, in effect defines a programme of further research, contextualises it and signposts how it may be pursued. We are, nevertheless, conscious that some of the signposts might be missing and others could be clearer, which we have endeavoured to address through additional referencing and clarifications in the text. 

**In response to specific comments:**

 In the section entitled 'Societal Considerations' we have revised the text to make it more precise and added supporting references to address the reviewer’s concern about the vagueness of the claims.

 In the section on ‘Taking Responsibility’ we have now added content to highlight the rich array of interactions that underlie behaviours not acceptable to the scientific community and areas to focus on when attempting to minimise such behaviours.

 The review questions the value system that the paper implicitly references. The value system used in China to appraise what He Jiankui did is a standard that is also widely accepted by Western countries, although this is not meant to imply that it is a “Western standard”. We have identified political, cultural and economic aspects that make the implementation of such standards difficult, but we did not imply that China is “alien” or “wide east” to an international value-system.

 In relation to those who failed to speak out against He Jiankui, the paper points out that mechanisms are lacking on several sides: local oversight is lax or lacking; and, there is no global mechanism that could help rectify lax or lacking local oversight. He Jiankui’s experiment could happen because of deficiencies on all sides.

 We remain grateful for the attention and advice of the reviewers and hope that the revisions have addressed their concerns satisfactorily.
